# HIV-1 *Gag-Pol* Sequences from Ugandan Early Infections Reveal Sequence Variants Associated with Elevated Replication Capacity

**DOI:** 10.3390/v13020171

**Published:** 2021-01-23

**Authors:** Anne Kapaata, Sheila N. Balinda, Rui Xu, Maria G. Salazar, Kimberly Herard, Kelsie Brooks, Kato Laban, Jonathan Hare, Dario Dilernia, Anatoli Kamali, Eugene Ruzagira, Freddie Mukasa, Jill Gilmour, Jesus F. Salazar-Gonzalez, Ling Yue, Matthew Cotten, Eric Hunter, Pontiano Kaleebu

**Affiliations:** 1 Medical Research Council, UVRI & LSTHM Uganda Research Unit, Plot 51–59, Entebbe, Uganda; Anne.Kapaata@mrcuganda.org (A.K.); Sheila.Balinda@mrcuganda.org (S.N.B.); lupita.g.salazar12@gmail.com (M.G.S.); Laban.Kato@mrcuganda.org (K.L.); Eugene.Ruzagira@mrcuganda.org (E.R.); Freddie.Kibengo@mrcuganda.org (F.M.); j.sal.gonz.3@gmail.com (J.F.S.-G.); Pontiano.Kaleebu@mrcuganda.org (P.K.); 2 Emory University, Atlanta, GA 30322, USA; rui.xu2@emory.edu (R.X.); kherard3@gmail.com (K.H.); kelsie.brooks@emory.edu (K.B.); ddilern@emory.edu (D.D.); lyue2@emory.edu (L.Y.); ehunte4@emory.edu (E.H.); 3 Imperial College London, London SW7 2AZ, UK; jhare@iavi.org (J.H.); JGilmour@iavi.org (J.G.); 4 International AIDS Vaccine Initiative (IAVI), New York, NY 10004, USA; 5 IAVI, Nairobi 00202, Kenya; AKamali@iavi.org; 6 Centre for Virus Research, MRC-University of Glasgow, Glasgow G61 1QH, UK

**Keywords:** HIV-1, Uganda, recombinant, *Gag-Pol*, protein domains

## Abstract

The ability to efficiently establish a new infection is a critical property for human immunodeficiency virus type 1 (HIV-1). Although the envelope protein of the virus plays an essential role in receptor binding and internalization of the infecting virus, the structural proteins, the polymerase and the assembly of new virions may also play a role in establishing and spreading viral infection in a new host. We examined Ugandan viruses from newly infected patients and focused on the contribution of the *Gag-Pol* genes to replication capacity. A panel of *Gag-Pol* sequences generated using single genome amplification from incident HIV-1 infections were cloned into a common HIV-1 NL4.3 pol/env backbone and the influence of *Gag-Pol* changes on replication capacity was monitored. Using a novel protein domain approach, we then documented diversity in the functional protein domains across the *Gag-Pol* region and identified differences in the Gag-p6 domain that were frequently associated with higher in vitro replication.

## 1. Introduction

During early HIV-1 infection, viremia increases rapidly, reaching a peak within weeks of infection, then drops to a level (the set point viral load or SPVL) that can remain stable over months to years of asymptomatic infection [1]. High SPVL is a predictor of faster disease progression [2]. The determinants of SPVL are complex and involve the host’s immune system as well as properties of the infecting virus and have been a matter of intensive research. SPVL and viral control vary by infecting subtype, with subtype A associated with control [3,4]. Subtype D HIV-1 infections have an increased frequency of CXCR4 co-receptor usage [5,6] and faster CD4+ T cell decline [7], which could account for the more aggressive clinical course HIV-1 subtype D infections than subtype A in sub-Saharan Africa [6,8,9,10,11].

Several studies report that the initial viruses establishing new HIV-1 infections may be important determinants of SPVL [12] and disease progression [13]. High viral replicative capacity (VRC) of transmitted HIV-1 among subtype C viruses has been associated with faster progression to disease [14,15]. Baalwa suggested that early subtype D viruses replicate more efficiently than subtype A [16] and subtype C viruses have lower VRC compared to other subtypes [17,18,19]. We asked if there were differences in VRC among Ugandan HIV-1 early viruses of subtypes A and D and their recombinants and set out to identify virus sequence features that might account for differences in VRC. The HIV-1 *gag* and *pol* genes are among the most conserved of the HIV-1 genome and in subtype C viruses appear to drive replication capacity and clinical outcomes [14,20]. Moreover, *Gag-Pol* chimeric viruses were shown to display similar VRC as the full-length HIV-1 genomes from which they were derived, supporting the idea that the *Gag-Pol* region was a major determinant of VRC. A large analysis of the *Gag-Pol* region from East African subtypes supported a hierarchy of inter-subtype recombinants replicating more highly in vitro than subtype D, which was in turn higher than subtypes A or C and identified changes in the Gag-p6 region that may play an important role among these chronically infected individuals [21]. Insertions in Gag-p6 are associated with increased replication as well as cooperation with protease resistance mutations [22,23,24]. Our study cohort consisted of HIV-seronegative individuals in the International AIDS Vaccine Initiative protocol C (IAVI protocol C) HIV epidemiology cohorts [25,26] who had been followed until seroconversion with frequent sampling intervals that allowed us to identify the virus near the time of transmission. We report here the molecular features of the *Gag-Pol* region of a set of these viruses and the contribution of these features to VRC. The results are important for determining the dynamics of HIV in human populations from East Africa where subtypes A, D and A/D recombinants predominate and may help identify sequence features associated with transmitted variants of distinct subtypes.

## 2. Materials and Methods

### 2.1. Study Subjects

This was a laboratory-based study incorporated into a larger multi-center primary HIV-1 infection cohort (IAVI protocol C) through Clinical Research Centers in Uganda, Kenya, Rwanda, Zambia and South Africa [26]. The protocol C study objectives were to follow the immunologic, virologic and clinical parameters in HIV-infected volunteers with a date of infection that could be accurately defined. In this study, data and samples were obtained from Ugandan participants, all initially HIV negative. Individuals who seroconverted were enrolled in IAVI protocol C. All were heterosexual individuals at high risk from the general population and from HIV-1 sero-discordant couples. Participants who became newly infected (tested positive for p24-antigen ELISA or HIV antibody) were invited to enroll. The estimated date of HIV infection (EDI) was defined as the midpoint between the last negative and first positive HIV antibody test, 14 days before the first positive p24 antigen test, 10 days before the first positive viral load test in the absence of p24 antigen or rapid HIV antibodies or the date of a self-reported high-risk exposure event. All participants were seen monthly until 3 months after EDI, then quarterly until 24 months and semi-annually thereafter. This study utilized protocol C stored plasma samples from 60 participants within 90 days post-EDI. The study received ethical approvals from the Uganda National Council of Science and Technology (REF: HS 108) on 8 February 2006, as well as from the UVRI Ethics Committee (REF: GC 127) on 9 December 2005. Study participants had consented to their samples being stored and used for future studies after approval by the relevant ethics committees.

### 2.2. Amplification and Sequencing Of Transmitted Virus for Identification of Early Gag-Pol Sequences

Viral RNA was isolated from 140 µL plasma using a QIA-amp Viral RNA Mini Kit (Qiagen Inc, Valencia, CA, USA). RNA was either frozen at −80 °C or immediately used to synthesize cDNA using SuperScript IV (Invitrogen, Ljubljana, Slovenia). Using a reverse primer 5FIV-R1 (5′-CTYTTTCTCCTGTATGCAGACCCC-3′; nucleotides 5272 to 5249 of the HXB2 sequence), cDNA was generated that served as a template to amplify a 5 kb 5′ half viral genome fragment spanning the *Gag-Pol* region. For single genome amplification (SGA), the cDNA was serially diluted in replicates of eight and subjected to nested PCR amplification with HIV-specific primers: 5FIV-R1 and RVDA-F1 (5′-GGGTCTCTCTDGTTAGACCAGAT-3′) for 1st round PCR and RVDA-F1 and 5FVR22 (5′-CCTAGTGGGATGTGTACTTCTGAAC-3′) for second round PCR. cDNA dilutions that yielded >30% PCR positive wells were retested in 96-well plates to identify a dilution where <30% of wells were positive for amplification products; these procedures and primers have been previously described in detail [27]. To ensure amplification from single molecules and avoid in vitro PCR artefacts, 8–10 SGA amplicons were generated per patient and these were sequenced using di-deoxy sequencing technology (Applied Biosystems 3500), aligned and analyzed using Sequencher and Geneious software to infer an early infection consensus sequence. HIV-1 subtype classification was done using the REGA (http://hivdb.stanford.edu/), the Recombination Identification Program (RIP) (http://www.hiv.lanl.gov/content/sequence/RIP/RIP.html) and jpHMM programs (GOBICS; University of Göttingen) [28,29,30] (Table 1). The jpHMM tool (http://jphmm.gobics.de/submission_hiv) was used to obtain recombination breakpoints, and the recombinant HIV-1 drawing tool from Los Alamos National Laboratories (https://www.hiv.lanl.gov/content/sequence/DRAW_CRF/recom_mapper.html) was used to generate the recombinant breakpoint maps.

### 2.3. Generation of Gag-Pol-NL4.3 Chimera Infectious Clones

The *Gag-Pol* amplicons were re-amplified with nested PCR with primers 5GagF:5′-TAGAAGGAGAGAGATGGGTGCGAG-3′ and POL_REV1 5′-CCATGTGTTAATCCTCATCCTGTC-3′ and cloned into an NL4.3 provirus backbone using the infusion homologous recombination method (Clontech Takara kit, Krakow, Poland). HIV-1 *Gag-Pol* infectious chimeric virus was packaged by transfection of 293T cells with pro-viral plasmids and titrated using a TZM-bl indicator cell assay, as described [12].

### 2.4. In Vitro Assay for HIV-1 Replicative Capacity

To assess the VRC of *Gag-Pol* NL4.3 chimeras, 5 × 10^5^ GXR25 cells [31] were infected at a multiplicity of infection (MOI) of 0.05. GXR25 cells and chimeric viruses were incubated with 5 µg/mL polybrene at 37 °C for 3 h, washed 5× with complete Roswell Park Memorial Institute 1640 medium (RPMI) and plated into 24-well plates. Cells were split 1:2 to maintain confluency by replacement with an equal amount of fresh media. Viral supernatants from days 2, 4, 6, 8 and 10 [32] and virions were quantified using a ^33^P-labeled reverse transcriptase assay and the colorimetric assay, as described below. The optimal window for logarithmic growth was determined to be between days 2–6. Replication capacity values were generated by dividing the area under the curve (AUC) for days 2–6 of the chimeric viruses by the AUC of the NL4.3 wildtype after subtracting the negative control [14]. Two independent *Gag-Pol* NL4.3 chimera clones per participant were run to confirm cloning fidelity.

### 2.5. Quantification of HIV-1 Reverse Transcriptase Using Radioactive and Colorimetric Assays

Culture supernatant aliquots from infected cells were added to a reverse transcriptase (RT) PCR master mix and incubated at 37 °C for 2 h; then the RT-PCR product was blotted onto DE-81 paper and allowed to dry. Blots were washed 5× with Saline sodium citrate buffer (SSC) and 3 times with 90% ethanol, allowed to dry and exposed to a phosphoscreen overnight. Counts were read using a Cyclone Phosphorimager [32]. The reverse transcriptase (RT) assay and colorimetric assay take advantage of the ability of reverse transcriptase to synthesize DNA using the hybrid poly (A) × oligo (dT) 15 as a template and primer. It avoids the use of [3H]- or [32P]-labeled nucleotides that are employed in standard RT assays. In place of radiolabeled nucleotides, digoxigenin- and biotin-labeled nucleotides in an optimized ratio are incorporated into the same DNA molecule by the RT activity. The detection and quantification of the synthesized DNA as a parameter for RT activity follows a sandwich ELISA protocol: biotin-labeled DNA binds to the surface of streptavidin-coated microplate modules. In the next step, an antibody to digoxigenin, conjugated to peroxidase (anti-DIG-POD), is added and bound to the digoxigenin-labeled nucleotides (licensed by Institut Pasteur). In the final step, the peroxidase substrate ABTS is added. The peroxidase enzyme catalyzes the cleavage of the substrate to produce a colored reaction product. The absorbance of the samples was determined using a microplate (ELISA) reader and was directly correlated to the level of RT activity in the sample using the manufacturer’s instructions (Sigma-Aldrich, Munich, Germany content version May 2016).

### 2.6. Protein Domain Methods

For the initial analysis, the encoded Pfam domains were identified using HMMER-3.2.1 [33] (http://hmmer.org/) with the Pfam database (Pfam 32.0 September 2018, (http://pfam.xfam.org/) [34]. For each sequence, all open reading frames ≥75 amino acids were determined from both reading strands and examined for Pfam content. A domain hit was retained if the domain i-Evalue was <0.0001. Details of each domain instance were gathered including position in query genome, length, domain i-Evalue and bit score. For the analysis in Figure 5, all full or nearly full HIV-1 genomes were retrieved from GenBank using the query (txid11676[Organism] AND 8000[SLEN]:11000[SLEN]) and HIV-1 subtype classification was performed using the KAMERIS tool [35].

### 2.7. Additional

The *Gag-Pol* sequences described here have been deposited in GenBank with the accession numbers MT027065-MT027082, MW316895-MW316901, MW316906-MW316908, MW316914, MW316916, MW316920 and MW316924.

## 3. Results

### 3.1. Participant and Virus Characteristics

**Figure 1:**
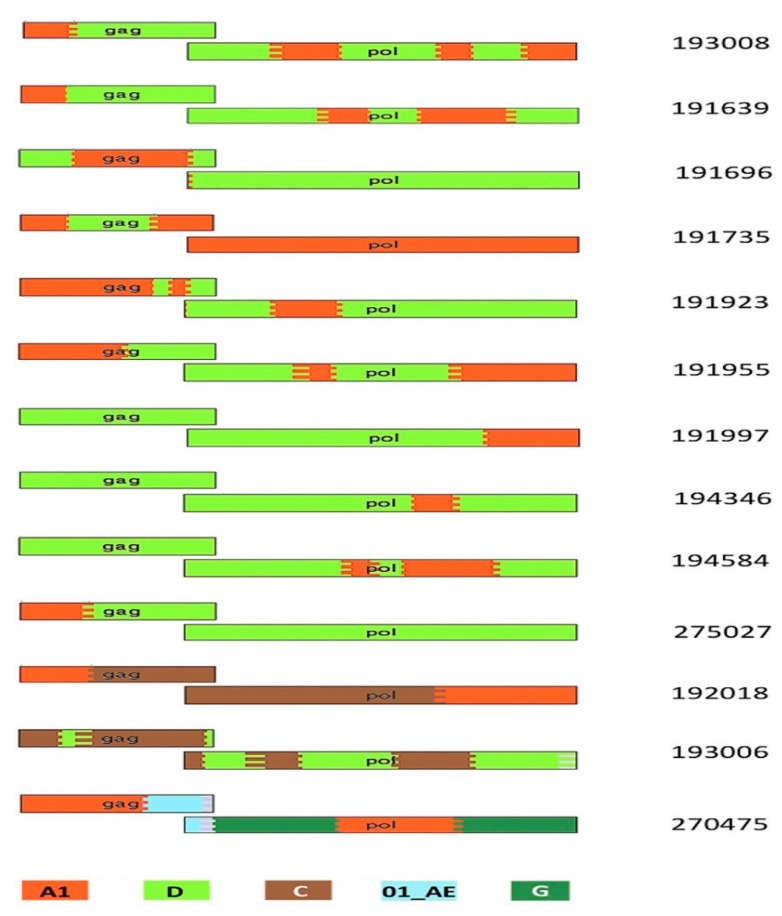
HIV-1 breakpoint map showing the recombination patterns across the 13 recombinants. This was generated using the jpHMM website and recombinant HIV-1 drawing tool from the LANL website as described in Materials and Methods. The key to colors in the figure: red as A1, light green as D, brown as C, dark green as G and light blue as 01_AE.

Thirty-two Ugandan protocol C participants had sequences successfully cloned from early samples drawn within 90 days of EDI and had their VRC characterized. Table 1 shows the participants’ characteristics. Three analysis tools, REGA, RIP and jpHMM [28,29,30] were used to assign subtypes and identify possible recombinants. We observed 6 with subtype A1, 13 with subtype D and 13 inter-subtype recombinants. The recombinants identified were A1D (10), A1C (1), CD (1) and a complex recombinant of subtypes E, F1, G and A (1) (Table 1, Figure 1).

### 3.2. Gag-Pol-NL4.3 Chimeras Showed a Range of Replicative Capacities

**Figure 2:**
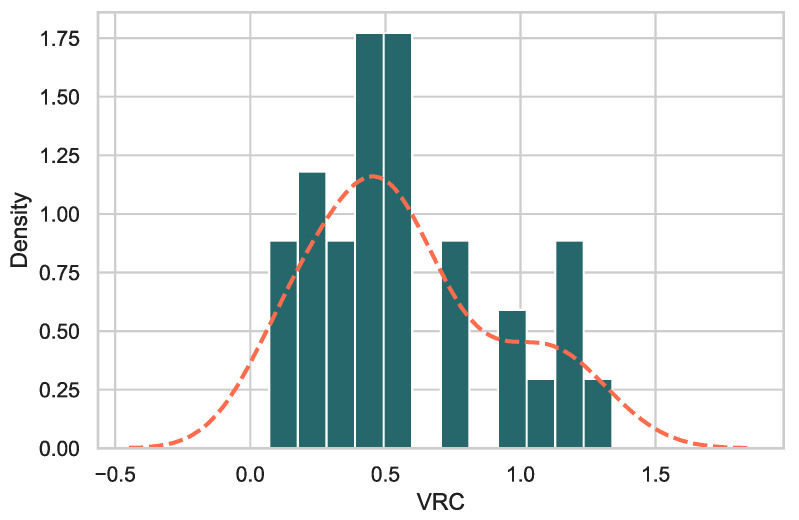
Histogram of VRC values across the 32 samples. The orange dashed line indicates a smoothed kernel density estimation (as implemented in Seaborn) of VRC values.

VRC was measured using *Gag-Pol* chimeras of early virus *Gag-Pol* cloned into an NL4.3 clone backbone [20,32]. The normalized VRC values of the chimeras for days 2–6 (logarithmic growth phase of these viruses) relative to wildtype NL4.3 ranged from 0.07–1.34 (Figure 2) The viral replicative capacity scores appeared to be biphasic, and accordingly, we used two groups (LowVRC ≤ 0.8 and HighVRC ≥ 0.8). The results demonstrate that replacement with a novel *Gag-Pol* region can have measurable effects on the ability of the virus to replicate in cell culture. When sequences were arranged by VRC (Table 1), the subtype A1 sequences show the lowest VRC values while subtype D, followed by the recombinants, show higher VRC values. The subtype of the Gag-P6 region within each sequence (see Table 1) shows a pattern, with higher VRC values found in sequences with non-A1 Gag-P6 (Table 1) and the highest VRCs found in viruses with more complex Gag-p6 regions.

### 3.3. There Was No Difference in Set Point Viral Load, CD4^+^ T Cell Count Decline and Subtypes

**Figure 3:**
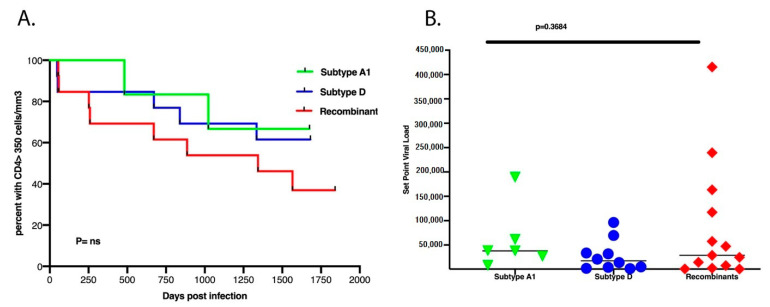
(**A**) There was no difference between the subtype classification of A1, D or the recombinants and the time taken for CD4^+^ T cell counts to decline to 350 cells/µL over the first 5.5 years of infection. (**B**) There was no difference between the subtypes and set point viral load.

Previous studies have documented the importance of the transmitted/founder (T/F) virus genotype in determining HIV-1 subtype B and C SPVL [36,37]. However, we observed no statistical correlation between the replication capacity of the *Gag-Pol* NL4.3 chimera and SPVL in this cohort of subtype A1, D and A1D recombinants (Figure 3B). The time taken for the CD4+ cell count to drop to less than 350 cells/µL between subtypes A1, D and recombinants also showed no statistical difference (Figure 3A).

### 3.4. Protein Domain Diversity of Gag-Pol Regions

**Figure 4:**
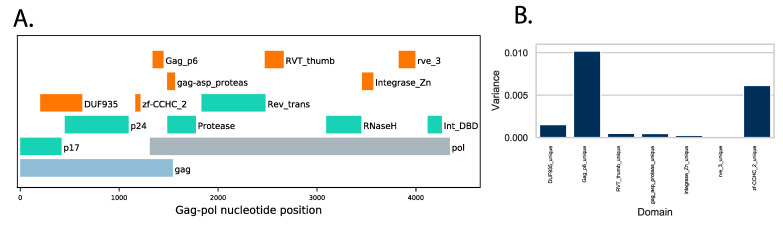
(**A**) Protein domains with variation across a query set of 32 HIV-1 *Gag-Pol* sequences. The HIV-1 *Gag-Pol* region is depicted, with open reading frames for gag (light blue) and pol (gray) indicated. The positions of HIV-1 Pfam domains with low variation (green) and the seven higher variation domains used here are colored in orange. (**B**) Variance of domain bit scores across the set of 32 query sequences.

To gain information about changes in viral protein functions associated with and perhaps influencing replication capacity, we used Pfam profile hidden Markov models (profile HMMs) to document differences in functional protein domains encoded by the viruses. Profile HMMs provide a statistical description of protein domains or cleavage sites and can be used to identify domains as well as to document changes in domain sequences relative to a reference set [34,38]. The functional domains of HIV-1 are well studied and provide a good starting point to identify protein motifs whose variation might influence virus replication. The 13 domains from the HIV-1 *Gag-Pol* region are described by Pfam, and preliminary results showed that seven domains (DUF935, zf-CCHC_2, Gag-P6 in the gag protein and gag_asp_proteas, RVT_thumb, integrase_Zn, rve_3 in the Pol protein, marked in green and orange in Figure 4A) showed variation in the set of 32 sequences (Figure 4B).

### 3.5. Variation of Gag-Pol Domains Linked to Elevated VRC

Using the Pfam domains [36] found in HIV-1 domains as guides, we prepared custom domains based on alignments from 391 subtype A1 complete genomes found in GenBank (see Section 2.7). Using A1 as the reference domain set allowed us to detect differences in the query sequences from the A1 type domains. For each of the 32 query sequences, the instances of the seven domains within the query sequences were identified and their domain bit scores (a measure of the distance of the query from the reference Pfam domain) were collected. The major contributors to variation were the Gag-P6 domain and the zinc finger CCHC domain, although modest changes were observed in the other domains (Figure 4B).

**Figure 5:**
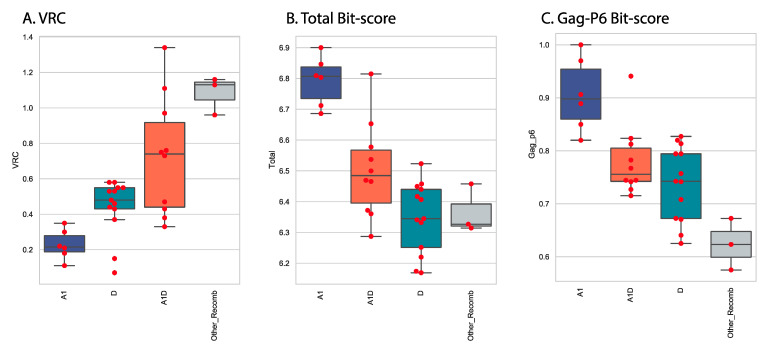
VRC, total domain scores and Gag-p6 domain bit scores by subtype. Scores for the 32 sequences were plotted as a function of the subtype category. For each panel, the median value is indicated by a horizontal line within the box, the top and bottom of the box indicate the interquartile range, individual values are indicated by red markers. Intergroup p values (Welch’s *t*-test) showed that all pairs were different (A1_VRC, D_VRC, *p* value = 0.0017; A1_VRC, A1D_VRC, *p* value = 0.0010; A1_VRC, Other_Recomb_VRC, *p* value = 0.0008; D_VRC, A1D_VRC, *p* value = 0.0281; D_VRC, Other_Recomb_VRC, *p* value = 0.0007; A1D_VRC, Other_Recomb_VRC, *p* value = 0.0151). (**A**) VRC vs. subtype, (**B**) Total domain bit-score vs subtypes, (**C**) Gag-P6 bitscore vs subtype.

Stratifying the *Gag-Pol* sequences into four subtype categories (A1, D, A1D and Other_recombinants) revealed important patterns (Figure 3). In vitro replication as measured by VRC was clearly different across the four groups, with the non-recombinant groups A1 and D showing lower VRC than the recombinants A1D and Other_Recombinants (CD, A1C, A1AEF) (Figure 5A). Combined total Pfam bit scores of all seven domains were calculated as a measure of how different the sequences were from the subtype A1 reference set. When total scores were compared across the four groups, the reverse pattern was seen, with the A1 sequences showing the highest scores (as expected, they were closest to the subtype A1 reference set) and the other groups showing more distance from subtype A1 sequences (Figure 5B). Within the domains analyzed, the major contribution to the distance score was in the Gag-P6 domain and, accordingly, the Gag-p6 scores showed a similar pattern to the total score (Figure 5C).

### 3.6. Protein Changes in Gag-P6 Region

**Figure 6:**
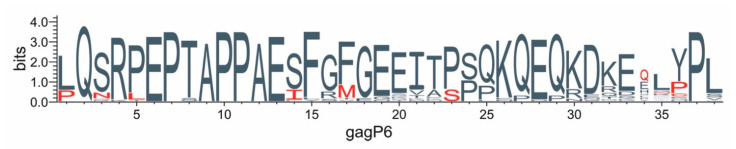
Protein changes in Gag-P6 region. The amino acid sequence of the Gag-P6 domains from the 32 sequences were aligned and a sequence logo was generated using Weblogo3 [39]. Amino acids are indicated by a single letter code with the height of each letter stack indicating conservation at that position (measured in entropy bits, see [39]) and the height of the letter within the stack indicating the relative frequency of the amino acid at that position. Amino acids found only in the genomes with VRC ≤ 0.4 are indicated in red.

A sequence logo of the Gag protein alignment shows the positions and residues unique to the low VRC sequences (Figure 6). The first proline in the Gag-P6 motif is part of the protease cleavage site 5′ to the Gag-P6 and seven of the eight low VRC sequences have a proline at this site (cleavage site FP), while there is leucine (cleavage site FL) in the majority of the medium and high VRC sequences (Figure 4). Similarly, low VRC sequences have either a proline or cysteine at position 36 near to the carboxy-terminal cleavage site flanking the Gag-P6 domain. These changes to or from proline near essential protease cleavage sites are expected to alter the local secondary structure and may play important roles in determining the efficiency of Gag polyprotein processing.

### 3.7. Global Gag-P6 Domain Variation

**Figure 7:**
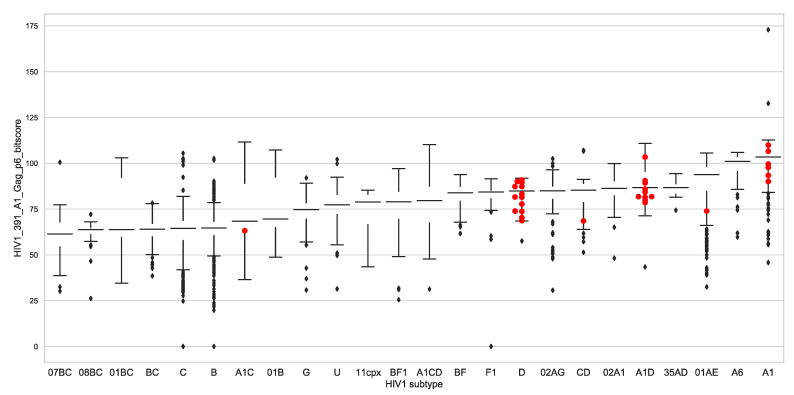
Comparing Gag-P6 bit scores for early infection sequences and all available HIV-1 genomes. All available HIV-1 genome sequences were retrieved from GenBank and classified by subtype using the KAMERIS tool [37]. Gag-P6 Pfam domains were identified and bit scores were gathered. The plot shows standard boxplots of the bit scores, stratified by the 25 HIV-1 subtypes identified in the set of genomes, with first interquartile range indicated by a colored box and the median value for each group indicated by a horizontal line. The Gag-P6 bit scores for the early sequences reported here are shown with red markers in their corresponding subtype. The counts of genomes by subtype were subtype B:7186, C:1750, 01_AE:1092, A1:414, 02_AG:244, BF1:225, G:178, 01B:168, BC:161, U:122, A1D:108, A1C:105, D:101, A6:98, F1:93, 02A1:83, CD:77, 01BC:72, O:71, BF:56, 07_BC:52, 08_BC:39, 11_cpx:29, A1CD:28, 35_AD:21, total: 12,573.

Because of the complexity of early infection identification, sequencing and VRC determination, our sample size was modest at 32 infections. To get an indication of the generality of Gag-P6 variation in HIV-1 biology, we expanded our analysis to include all available HIV-1 full genome sequences. We asked if the observed Gag-P6 domain variation occurred in HIV-1 genomes from chronic infections. To answer this question, all available HIV-1 complete genome sequences were retrieved from GenBank (12,571 genomes, 30 October 2019) and classified by subtype. The majority of the HIV-1 genome sequences in GenBank are expected to be derived from chronic infections due to acute infection (by definition) being time-limited and the complexity of obtaining acute infection samples. For all available near-full-length HIV genomes, subtypes were determined, the Gag-p6 Pfam bit scores were determined and for each subtype, a median Gag-p6 Pfam bit score was calculated. We then compared the 32 early Gag-p6 Pfam bit scores generated from the acute infection study to the median values for the GenBank set of 12,571 genomes (Figure 7). We found that 21 of the Gag-p6 bit scores fall below the median value for their corresponding subtype (showing greater protein distance from the subtype A1 reference) and 14 of 32 scores fell below the interquartile range, the normal range of variation found in viruses from chronic sequences (Figure 5). This shows increased variability (lower bit scores) in the Gag-p6 domains of early infection sequences relative to the Gag-p6 domains from chronic infections.

## 4. Discussion

In this study, we documented the VRC supported by *Gag-Pol* gene chimeras with NL4.3 viruses generated from 32 Ugandan adults with very early HIV infection. The study included the subtypes typically observed in Uganda, that is, subtype A, D and A1D recombinants. The recombinant breakpoints greatly varied among the 13 recombinants identified in this study, as shown in Figure 1. Our results indicate that the set of *Gag-Pol* genes described here support a range of VRCs, with some variants showing a higher VRC than that of the wildtype NL4.3. In general, subtype A1 had the lowest VRC, followed by subtype D, with inter-subtype recombinants having the greatest VRC. When looking at only the subtype classification of the Gag-p6 region (Figure 1), this is consistent with earlier reports of inter-subtype differences in disease progression where recombinants progressed fastest, followed by subtype D, with subtype A progressing the slowest [9,10,11]. Our study results are also consistent with earlier studies that showed inter-subtype recombinants having higher replicative fitness than pure subtypes [39,40] in West Africa. Another study in East African cohorts showed a similar trend of hierarchy of Gag protease-driven replication capacities, with subtypes A or C replicating less, followed by D, and inter-subtype recombinants replicating the most [21].

Increasing evidence indicates that in vitro VRC appears to be a strong indicator of HIV pathogenicity in the patient [14,20,41,42] Here, we observed that while there were differences in VRC between subtypes A, D and recombinant *Gag-pol*, there was no correlation between VRC and CD4+ cell count levels or viral load in the small number of patients examined (results not shown). There was, however, a trend where most high replicators progressed faster to CD4+ counts of less than 350 cells/µL in the first 5 years of infection, although this was not statistically significant. However, no trends or significant correlations between SPVL and VRC were observed (results not shown). This suggests that the VRC of the initial infecting strain may have limited impact on these important long-term markers of HIV pathogenesis.

To gain information about viral protein functions that might be associated with the observed differences in replication capacity, we monitored changes in the Pfam profile hidden Markov models found in these sequences to reveal differences in functional or defined protein domains in the *Gag-Pol* genes. Rather than categorizing VRC by general subtype, the domain analysis we performed provided a more detailed focus on changes in protein domains with functional attributes. Across the set of 32 sequences, there was variability in three domains in the Gag coding region: a domain of unknown function DUF935 in the amino terminal half of the protein, the zinc finger motif zf_CCHC_2 and the gag-p6 domain near the C-terminus and overlapping with the Pol coding region. Gag-p6 is a major phosphoprotein of HIV-1 that has been shown to play an important role when it comes to release of the virus from the infected cells [43]. The four viruses with the highest VRCs showed the greatest level of variety in the Gag-p6 domain (lowest HMM bit score), suggesting that changes in this domain may influence viral replication. Two sequences had insertions related to a PYxE insert previously observed in subtype C viruses with elevated virulence [44]. The PYxE motif may be involved in the ALIX (ALG-2 (apoptosis-linked gene 2)-interacting protein X)-mediated virus release pathway [45] and recently the insertion of this tetrapeptide has been implicated in the restoration of Gag binding to ALIX with enhanced viral fitness in the presence or absence of lopinavir and tenofovir alafenamide antiretroviral drugs [23].

The HIV-1 nucleocapsid protein carries two zinc fingers and is located at the C-terminus of Gag, trailed by the p6 domain. The zf-CCHC_2 domain is one of the two zinc finger domains in the Gag nucleocapsid protein and both are required for protein localization, genomic RNA binding and encapsidation [46,47,48]. All zinc finger changes or mutations in one study were shown to negatively impact on virus replication and maturation [49]. The gag-p6 domain is needed for particle budding, during which the viral particles pinch off from the cellular membrane [50]. The p6 domain additionally contains proline-rich and di-leucine areas, which are the target of the cellular proteins Tsg101 and Alix, respectively, which are involved in the cellular class E protein sorting pathway and HIV-1 budding machinery [51,52].

We asked if the observed Gag-P6 domain variants were unique to incident viruses or if similar variation can be observed in HIV-1 genomes derived from chronic infection. We examined the Gag-P6 domain from all available full or nearly full genomes from GenBank (Figure 5). Comparing the Gag-P6 bit scores (a measure of the distance of the query sequence to the reference domain) to median scores for each HIV-1 subtype showed that 21 of the early infection sequences had Gag-P6 bit scores below the median value for their subtype (Figure 5). Lower Gag-P6 bit scores indicate greater variation from the A1 reference domain, thus there is a tendency for changes in the Gag-P6 sequences. The Gag-P6 region is emerging as an important determinant of HIV-1 replication [23,44,45]. Although it seems unlikely that a Gag-P6 variant unique to early infection sequences exists, the increased variation in this site observed in this small set of 32 patients is consistent with the domain playing a role in transmission. It is also notable that additional changes were observed in six other *Gag-Pol* domains (Figure 2 and these may cooperate with the Gag-p6 alterations in viruses associated with transmission.

The first proline in the Gag-P6 motif is part of the protease cleavage site 5′ to Gag-P6 and seven of the eight low VRC sequences have a proline at this site (cleavage site FP) while there is leucine (cleavage site FL) in the majority of the medium and high VRC sequences (Figure 4). Similarly, low VRC sequences have either a proline or cysteine at position 36 near to the carboxy-terminal cleavage site flanking the Gag-P6 domain. These changes to or from proline near essential protease cleavage sites may play important roles in determining the efficiency of Gag polyprotein processing, which in turn influences the viral packaging and viral load and perhaps plays an important role in establishing early infection. It should be noted that the proline to serine or proline to leucine coding changes require only a 1 nt change and may account for the diversity observed at this site. One can speculate that as infections progress to a chronic stage, it may be useful to reduce viral loads to avoid immune responses and simple amino acid switches might be involved.

Our study had some limitations. The effort required for SGA cloning limited the number of sequences available. The VRC measurement is a simplified virus replication in the absence of immune responses and the measurements were performed using a query *Gag-Pol* sequence within an HXB2 backbone virus. This potentially misses more complex interactions between the *Gag-Pol* region and the rest of the virus. However, despite the modest sample size, we were still able to observe strong differences in VRC by HIV-1 subtype. The samples were obtained in 2006–2011 and HIV-1 evolution has continued. However, the global analysis shown in Figure 5 included more recent sequence data up to December 2019 and the Gag-p6 variations we observed in the set of 32 early infection sequences appeared to be representative of the entire HIV-1 epidemic.

In conclusion, the current study has revealed crucial features of the HIV-1 *Gag-Pol* region, especially the Gag-p6 domain that influences viral replicative capacity and may play a role in establishing new HIV-1 infections.

## Figures and Tables

**Table 1:** Participants’ characteristics of 32 early infected Ugandans.

Participant ID	Subtype ^a^	VRC Score	Participant Gender	Participant Age	Days Post-EDI	Set Point Viral Load	Visit CD4 Count (Cells/µL)
191084	A1	0.21	Female	29	27	61,309	777
191637	A1	0.18	Male	31	85	26,595	634
191734	A1	0.35	Male	44	67	38,081	462
191918	A1	0.11	Male	22	56	8005	878
194180	A1	0.3	Male	46	59	37,767	438
270909	A1	0.22	Male	41	50	189,054	536
Mean *	--	--	--	35.5	57.33	60,135	621
191996	D	0.53	Female	37	55	1599	806
192002	D	0.37	Female	27	50	2122	569
194020	D	0.55	Male	33	36	ND	783
194037	D	0.55	Male	34	51	33,550	531
194374	D	0.43	Male	33	35	31,954	401
194535	D	0.48	Male	39	47	ND	281
194603	D	0.58	Female	33	52	ND	887
194604	D	0.53	Female	35	44	69,512	677
270015	D	0.15	Male	58	11	14,064	355
270535	D	0.07	Male	31	73	5197	796
275026	D	0.46	Female	21	51	96,368	277
275031	D	0.44	Male	31	25	4260	795
194065	D	0.58	Male	41	42	20,890	470
Mean **	--	--	--	34.85	44	27,952	587
193008	A1D	1.34	Male	27	23	57,464	798
191639	A1D	0.47	Male	50	50	117,145	1149
191696	A1D	0.33	Male	29	50	239,477	398
191735	A1D	0.38	Male	22	56	2482	754
191923	A1D	1.11	Female	31	55	28,929	242
191955	A1D	0.76	Female	39	23	696	997
191997	A1D	0.97	Male	31	57	1002	764
194346	A1D	0.75	Male	29	31	415,426	346
194584	A1D	0.43	Female	33	25	7780	580
275027	A1D	0.73	Female	22	61	163,395	651
192018	A1C	0.96	Male	22	29	24,769	352
193006	CD	1.16	Male	24	52	47,355	478
270475	01AE	1.13	Female	28	43	14,528	531
Mean ***	--	--	--	29.77	42.69	86,188	618

The most frequent subtype with the final designation; ^a^ final subtype designation was taken from the majority classification of the three methods using *Gag-Pol* sequences. The 32 *Gag-Pol* sequences were analyzed with the following HIV subtype assignment tools: RIP (http://www.hiv.lanl.gov/tent/sequence/RIP/RIP.html), REGA at http://hivdb.stanford.edu/ and jpHMM at http://jphmm.gobics.de. Mean * denotes the mean for subtype A1, Mean ** denotes subtype D and Mean *** denotes recombinants. EDI—estimated date of HIV infection, VRC—viral replicative capacity.

## Data Availability

Data are available in GenBank (see Section 2.7).

## References

[1] Fiebig E.W., Wright D.J., Rawal B.D., Garrett P.E., Schumacher R.T., Peddada L., Heldebrant C., Smith R., Conrad A., Kleinman S.H. (2003). Dynamics of HIV Viremia and Antibody Seroconversion in Plasma Donors: Implications for Diagnosis and Staging of Primary HIV Infection. Aids.

[2] Hansmann A., Schim van der Loeff M.F., Kaye S., Awasana A.A., Sarge-Njie R., O’Donovan D., Ariyoshi K., Alabi A., Milligan P., Whittle H.C. (2005). Baseline Plasma Viral Load and CD4 Cell Percentage Predict Survival in HIV-1- and HIV-2-Infected Women in a Community-Based Cohort in The Gambia. J. Acquir. Immune Defic. Syndr..

[3] Price M.A., Rida W., Kilembe W., Karita E., Inambao M., Ruzagira E., Kamali A., Sanders E.J., Anzala O., Hunter E. (2019). Control of the HIV-1 Load Varies by Viral Subtype in a Large Cohort of African Adults With Incident HIV-1 Infection. J. Infect. Dis..

[4] Prentice H.A., Price M.A., Porter T.R., Cormier E., Mugavero M.J., Kamali A., Karita E., Lakhi S., Sanders E.J., Anzala O. (2014). Dynamics of Viremia in Primary HIV-1 Infection in Africans: Insights from Analyses of Host and Viral Correlates. Virology.

[5] Huang W., Eshleman S.H., Toma J., Fransen S., Stawiski E., Paxinos E.E., Whitcomb J.M., Young A.M., Donnell D., Mmiro F. (2007). Coreceptor Tropism in Human Immunodeficiency Virus Type 1 Subtype D: High Prevalence of CXCR4 Tropism and Heterogeneous Composition of Viral Populations. J. Virol..

[6] Kaleebu P., Nankya I.L., Yirrell D.L., Shafer L.A., Kyosiimire-Lugemwa J., Lule D.B., Morgan D., Beddows S., Weber J., Whitworth J.A.G. (2007). Relation between Chemokine Receptor Use, Disease Stage, and HIV-1 Subtypes A and D: Results from a Rural Ugandan Cohort. J. Acquir. Immune Defic. Syndr..

[7] Kiwanuka N., Robb M., Laeyendecker O., Kigozi G., Wabwire-Mangen F., Makumbi F.E., Nalugoda F., Kagaayi J., Eller M., Eller L.A. (2010). HIV-1 Viral Subtype Differences in the Rate of CD4+ T-Cell Decline among HIV Seroincident Antiretroviral Naive Persons in Rakai District, Uganda. J. Acquir. Immune Defic. Syndr..

[8] Baeten J.M., Chohan B., Lavreys L., Chohan V., McClelland R.S., Certain L., Mandaliya K., Jaoko W., Overbaugh J. (2007). HIV-1 Subtype D Infection Is Associated with Faster Disease Progression than Subtype A in Spite of Similar Plasma HIV-1 Loads. J. Infect. Dis..

[9] Kiwanuka N., Laeyendecker O., Robb M., Kigozi G., Arroyo M., McCutchan F., Eller L.A., Eller M., Makumbi F., Birx D. (2008). Effect of Human Immunodeficiency Virus Type 1 (HIV-1) Subtype on Disease Progression in Persons from Rakai, Uganda, with Incident HIV-1 Infection. J. Infect. Dis..

[10] Kaleebu P., French N., Mahe C., Yirrell D., Watera C., Lyagoba F., Nakiyingi J., Rutebemberwa A., Morgan D., Weber J. (2002). Effect of Human Immunodeficiency Virus (HIV) Type 1 Envelope Subtypes A and D on Disease Progression in a Large Cohort of HIV-1–Positive Persons in Uganda. J. Infect. Dis..

[11] Ssemwanga D., Nsubuga R.N., Mayanja B.N., Lyagoba F., Magambo B., Yirrell D., Van der Paal L., Grosskurth H., Kaleebu P. (2013). Effect of HIV-1 Subtypes on Disease Progression in Rural Uganda: A Prospective Clinical Cohort Study. PLoS ONE.

[12] Prince J.L., Claiborne D.T., Carlson J.M., Schaefer M., Yu T., Lahki S., Prentice H.A., Yue L., Vishwanathan S.A., Kilembe W. (2012). Role of Transmitted Gag CTL Polymorphisms in Defining Replicative Capacity and Early HIV-1 Pathogenesis. PLoS Pathog..

[13] Wright J.K., Novitsky V., Brockman M.A., Brumme Z.L., Brumme C.J., Carlson J.M., Heckerman D., Wang B., Losina E., Leshwedi M. (2011). Influence of Gag-Protease-Mediated Replication Capacity on Disease Progression in Individuals Recently Infected with HIV-1 Subtype C. J. Virol..

[14] Claiborne D.T., Prince J.L., Scully E., Macharia G., Micci L., Lawson B., Kopycinski J., Deymier M.J., Vanderford T.H., Nganou-Makamdop K. (2015). Replicative Fitness of Transmitted HIV-1 Drives Acute Immune Activation, Proviral Load in Memory CD4+ T Cells, and Disease Progression. Proc. Natl. Acad. Sci. USA.

[15] Prado J.G., Prendergast A., Thobakgale C., Molina C., Tudor-Williams G., Ndung’u T., Walker B.D., Goulder P. (2010). Replicative Capacity of Human Immunodeficiency Virus Type 1 Transmitted from Mother to Child Is Associated with Pediatric Disease Progression Rate. J. Virol..

[16] Baalwa J., Wang S., Parrish N.F., Decker J.M., Keele B.F., Learn G.H., Yue L., Ruzagira E., Ssemwanga D., Kamali A. (2013). Molecular Identification, Cloning and Characterization of Transmitted/Founder HIV-1 Subtype A, D and A/D Infectious Molecular Clones. Virology.

[17] Abraha A., Nankya I.L., Gibson R., Demers K., Tebit D.M., Johnston E., Katzenstein D., Siddiqui A., Herrera C., Fischetti L. (2009). CCR5- and CXCR4-Tropic Subtype C Human Immunodeficiency Virus Type 1 Isolates Have a Lower Level of Pathogenic Fitness than Other Dominant Group M Subtypes: Implications for the Epidemic. J. Virol..

[18] Ariën K.K., Abraha A., Quiñones-Mateu M.E., Kestens L., Vanham G., Arts E.J. (2005). The Replicative Fitness of Primary Human Immunodeficiency Virus Type 1 (HIV-1) Group M, HIV-1 Group O, and HIV-2 Isolates. J. Virol..

[19] Ball S.C., Abraha A., Collins K.R., Marozsan A.J., Baird H., Quiñones-Mateu M.E., Penn-Nicholson A., Murray M., Richard N., Lobritz M. (2003). Comparing the Ex Vivo Fitness of CCR5-Tropic Human Immunodeficiency Virus Type 1 Isolates of Subtypes B and C. J. Virol..

[20] Ojwach D.B.A., MacMillan D., Reddy T., Novitsky V., Brumme Z.L., Brockman M.A., Ndung’u T., Mann J.K. (2018). Pol-Driven Replicative Capacity Impacts Disease Progression in HIV-1 Subtype C Infection. J. Virol..

[21] Kiguoya M.W., Mann J.K., Chopera D., Gounder K., Lee G.Q., Hunt P.W., Martin J.N., Ball T.B., Kimani J., Brumme Z.L. (2017). Subtype-Specific Differences in Gag-Protease-Driven Replication Capacity Are Consistent with Intersubtype Differences in HIV-1 Disease Progression. J. Virol..

[22] Neogi U., Engelbrecht S., Claassen M., Jacobs G.B., van Zyl G., Preiser W., Sonnerborg A. (2016). Mutational Heterogeneity in P6 Gag Late Assembly (L) Domains in HIV-1 Subtype C Viruses from South Africa. AIDS Res. Hum. Retrovir..

[23] van Domselaar R., Njenda D.T., Rao R., Sönnerborg A., Singh K., Neogi U. (2019). HIV-1 Subtype C with PYxE Insertion Has Enhanced Binding of Gag-P6 to Host Cell Protein ALIX and Increased Replication Fitness. J. Virol..

[24] Martins A.N., Waheed A.A., Ablan S.D., Huang W., Newton A., Petropoulos C.J., Brindeiro R.D.M., Freed E.O. (2016). Elucidation of the Molecular Mechanism Driving Duplication of the HIV-1 PTAP Late Domain. J. Virol..

[25] Kamali A., Price M.A., Lakhi S., Karita E., Inambao M., Sanders E.J., Anzala O., Latka M.H., Bekker L.-G., Kaleebu P. (2015). Creating an African HIV Clinical Research and Prevention Trials Network: HIV Prevalence, Incidence and Transmission. PLoS ONE.

[26] Amornkul P.N., Karita E., Kamali A., Rida W.N., Sanders E.J., Lakhi S., Price M.A., Kilembe W., Cormier E., Anzala O. (2013). Disease Progression by Infecting HIV-1 Subtype in a Seroconverter Cohort in Sub-Saharan Africa. Aids.

[27] Salazar-Gonzalez J.F., Salazar M.G., Keele B.F., Learn G.H., Giorgi E.E., Li H., Decker J.M., Wang S., Baalwa J., Kraus M.H. (2009). Genetic Identity, Biological Phenotype, and Evolutionary Pathways of Transmitted/Founder Viruses in Acute and Early HIV-1 Infection. J. Exp. Med..

[28] de Oliveira T., Deforche K., Cassol S., Salminen M., Paraskevis D., Seebregts C., Snoeck J., van Rensburg E.J., Wensing A.M.J., van de Vijver D.A. (2005). An Automated Genotyping System for Analysis of HIV-1 and Other Microbial Sequences. Bioinformatics.

[29] Schultz A.-K., Zhang M., Bulla I., Leitner T., Korber B., Morgenstern B., Stanke M. (2009). JpHMM: Improving the Reliability of Recombination Prediction in HIV-1. Nucleic Acids Res..

[30] Siepel A.C., Halpern A.L., Macken C., Korber B.T. (1995). A Computer Program Designed to Screen Rapidly for HIV Type 1 Intersubtype Recombinant Sequences. AIDS Res. Hum. Retrovir..

[31] Brockman M.A., Tanzi G.O., Walker B.D., Allen T.M. (2006). Use of a Novel GFP Reporter Cell Line to Examine Replication Capacity of CXCR4- and CCR5-Tropic HIV-1 by Flow Cytometry. J. Virol. Methods.

[32] Wright J.K., Naidoo V.L., Brumme Z.L., Prince J.L., Claiborne D.T., Goulder P.J.R., Brockman M.A., Hunter E., Ndung’u T. (2012). Impact of HLA-B*81-Associated Mutations in HIV-1 Gag on Viral Replication Capacity. J. Virol..

[33] Eddy S.R. (2011). Accelerated Profile HMM Searches. PLOS Comput. Biol..

[34] El-Gebali S., Mistry J., Bateman A., Eddy S.R., Luciani A., Potter S.C., Qureshi M., Richardson L.J., Salazar G.A., Smart A. (2019). The Pfam Protein Families Database in 2019. Nucleic Acids Res..

[35] Solis-Reyes S., Avino M., Poon A., Kari L. (2018). An Open-Source k-Mer Based Machine Learning Tool for Fast and Accurate Subtyping of HIV-1 Genomes. PLoS ONE.

[36] Yue L., Prentice H.A., Farmer P., Song W., He D., Lakhi S., Goepfert P., Gilmour J., Allen S., Tang J. (2013). Cumulative Impact of Host and Viral Factors on HIV-1 Viral-Load Control during Early Infection. J. Virol..

[37] Hollingsworth T.D., Laeyendecker O., Shirreff G., Donnelly C.A., Serwadda D., Wawer M.J., Kiwanuka N., Nalugoda F., Collinson-Streng A., Ssempijja V. (2010). HIV-1 Transmitting Couples Have Similar Viral Load Set-Points in Rakai, Uganda. PLoS Pathog..

[38] Crooks G.E. (2004). WebLogo: A Sequence Logo Generator. Genome Res..

[39] Konings F.A.J., Burda S.T., Urbanski M.M., Zhong P., Nadas A., Nyambi P.N. (2006). Human Immunodeficiency Virus Type 1 (HIV-1) Circulating Recombinant Form 02_AG (CRF02_AG) Has a Higher in Vitro Replicative Capacity than Its Parental Subtypes A and G. J. Med. Virol..

[40] Njai H.F., Gali Y., Vanham G., Clybergh C., Jennes W., Vidal N., Butel C., Mpoudi-Ngolle E., Peeters M., Ariën K.K. (2006). The Predominance of Human Immunodeficiency Virus Type 1 (HIV-1) Circulating Recombinant Form 02 (CRF02_AG) in West Central Africa May Be Related to Its Replicative Fitness. Retrovirology.

[41] Goepfert P.A., Lumm W., Farmer P., Matthews P., Prendergast A., Carlson J.M., Derdeyn C.A., Tang J., Kaslow R.A., Bansal A. (2008). Transmission of HIV-1 Gag Immune Escape Mutations Is Associated with Reduced Viral Load in Linked Recipients. J. Exp. Med..

[42] Brockman M.A., Schneidewind A., Lahaie M., Schmidt A., Miura T., Desouza I., Ryvkin F., Derdeyn C.A., Allen S., Hunter E. (2007). Escape and Compensation from Early HLA-B57-Mediated Cytotoxic T-Lymphocyte Pressure on Human Immunodeficiency Virus Type 1 Gag Alter Capsid Interactions with Cyclophilin A. J. Virol..

[43] Müller B., Patschinsky T., Kräusslich H.-G. (2002). The Late-Domain-Containing Protein P6 Is the Predominant Phosphoprotein of Human Immunodeficiency Virus Type 1 Particles. J. Virol..

[44] Aralaguppe S.G., Winner D., Singh K., Sarafianos S.G., Quiñones-Mateu M.E., Sönnerborg A., Neogi U. (2017). Increased Replication Capacity Following Evolution of PYxE Insertion in Gag-P6 Is Associated with Enhanced Virulence in HIV-1 Subtype C from East Africa. J. Med. Virol..

[45] Neogi U., Rao S.D., Bontell I., Verheyen J., Rao V.R., Gore S.C., Soni N., Shet A., Schülter E., Ekstrand M.L. (2014). Novel Tetra-Peptide Insertion in Gag-P6 ALIX-Binding Motif in HIV-1 Subtype C Associated with Protease Inhibitor Failure in Indian Patients. Aids.

[46] Gorelick R.J., Nigida S.M., Bess J.W., Arthur L.O., Henderson L.E., Rein A. (1990). Noninfectious Human Immunodeficiency Virus Type 1 Mutants Deficient in Genomic RNA. J. Virol..

[47] Gorelick R.J., Chabot D.J., Rein A., Henderson L.E., Arthur L.O. (1993). The Two Zinc Fingers in the Human Immunodeficiency Virus Type 1 Nucleocapsid Protein Are Not Functionally Equivalent. J. Virol..

[48] Gorelick R.J., Gagliardi T.D., Bosche W.J., Wiltrout T.A., Coren L.V., Chabot D.J., Lifson J.D., Henderson L.E., Arthur L.O. (1999). Strict Conservation of the Retroviral Nucleocapsid Protein Zinc Finger Is Strongly Influenced by Its Role in Viral Infection Processes: Characterization of HIV-1 Particles Containing Mutant Nucleocapsid Zinc-Coordinating Sequences. Virology.

[49] Grigorov B., Décimo D., Smagulova F., Péchoux C., Mougel M., Muriaux D., Darlix J.-L. (2007). Intracellular HIV-1 Gag Localization Is Impaired by Mutations in the Nucleocapsid Zinc Fingers. Retrovirology.

[50] Demirov D.G., Freed E.O. (2004). Retrovirus Budding. Virus Res..

[51] Strack B., Calistri A., Craig S., Popova E., Göttlinger H.G. (2003). AIP1/ALIX Is a Binding Partner for HIV-1 P6 and EIAV P9 Functioning in Virus Budding. Cell.

[52] von Schwedler U.K., Stuchell M., Müller B., Ward D.M., Chung H.-Y., Morita E., Wang H.E., Davis T., He G.-P., Cimbora D.M. (2003). The Protein Network of HIV Budding. Cell.

